# Exploring the Impact of the “RUEU?” Game on Greek Students’ Perceptions of and Attitudes to European Identity

**DOI:** 10.3389/fpsyg.2022.834846

**Published:** 2022-03-16

**Authors:** Athanassios Jimoyiannis, Elizabeth A. Boyle, Panagiotis Tsiotakis, Melody M. Terras, Murray S. Leith

**Affiliations:** ^1^Department of Social and Educational Policy, University of Peloponnese, Korinthos, Greece; ^2^School of Education and Social Sciences, University of the West of Scotland, Paisley, United Kingdom

**Keywords:** serious games, gamification, European identity, attitude change, university students

## Abstract

European identity is a complex, multi-faced and inherently imprecise concept relating to a range of socio-political and psychological factors. Addressing this topic in educational practice, particularly with respect to Higher Education students, constitutes a complex and open problem for research. This paper reports on an experimental study designed to explore the effectiveness of the educational game “RUEU?” in supporting university students in understanding the key socio-political issues regarding European identity. Quantitative data regarding Greek university students’ (*N* = 92) attitudes to European identity, before and after playing the game, were collected. Students’ performance of the game group (*N* = 46) was compared with that of a control group (*N* = 46) who explored the same issues about European identity through a tutor-guided discussion. The findings showed that both instructional interventions were effective but in different ways. The participants in the game-based group appeared more attached to the EU after the intervention and moved toward a more balanced description of their Greek and European identity. On the other hand, the students in the control group rated higher what “European identity” and being EU citizen means to them. The findings indicated the potential of game-based interventions to deliver not only academic content but also to promote students’ reflection and influence them at an attitudinal and emotional level.

## Introduction

European identity is becoming a relevant identity for people living in Europe, alongside their national identity. Although national and European identification are generally positively associated, the actual strength of this association varies across different European countries (Recchi and Salamońska, 2014; Lam and Katona, 2018; Jugert et al., 2019) with recent research suggesting that the development of national and European identity is related to people’s age, as well as their education level (Van der Zwet et al., 2020). Results from the Eurobarometer (European Commission, 2007) showed that adolescents (15–19 years) have more positive views about the EU than young adults (20–30 years). Lam and Katona (2018) reported stronger European identity among young Hungarian adolescents (13–15 year-olds) than 16–18 year-olds, while Citrin and Sides (2004) illustrated that more highly educated individuals are more likely to feel European. Moreover, European identity is also dependent on the socio-economic context of the participants; for example adolescents coming from lower and middle socio-economic levels showed stronger Hungarian vs. European identification (Lam and Katona, 2018). Data from students in Czech Republic and Germany, indicates that EU identity patterns are very similar across domains and countries (Jugert et al., 2021).

Cross-border mobility is associated with a stronger European identification, more positive attitudes toward the EU, and validates a multifaceted vision of European Union based on a range of dimensions (e.g., shared values, history, economic, and political) among European adolescents and young adults in eight countries (Mazzoni et al., 2018). International mobility has a positive impact on university students’ perceptions about European identity. This effect is clearly apparent in the development of European identity among Erasmus students, in terms of their personal development, cultural enhancement, international understanding, European consciousness, and feeling of belonging to Europe (Jacobone and Moro, 2015). However, a recent study in Spain, by Méndez García et al. (2021), revealed that only a small group of Andalusian students recognized a moderate or relatively strong feeling of European identity. The results also indicated that the students’ mobility and returnees from studying abroad displayed no differences in their perceptions of European identity compared to the students who had no opportunity to participate in international study programs.

Nevertheless, as a political and psychological notion, European identity is a complex, multi-faced and inherently uncertain concept. Literature suggested that to address this topic in educational practice we need to include a diversity of knowledge and values regarding both national and European identities. To achieve this objective, scholars have advocated alternative approaches and learning environments that promote students’ active experimentation and reflection on personal thinking about European identity, as well as understanding the views of other people. Educational gaming is a promising choice since games can support players in knowledge construction, and behavioral and attitude change in various domains by harnessing the engaging, playful and enjoyable features of serious games (Boyle et al., 2019a).

Previous research on educational interventions with regards to national and European identity, in particular among youth and Higher Education (HE) students in Europe, is rather limited (Boyle et al., 2021). To our knowledge, there is no study examining instructional interventions on university students’ development and reflection on their perceptions about European identity.

This study aims to contribute to this research domain by describing the empirical foundation of the effectiveness of the “*RUEU?*” (are you EU?) game (Boyle et al., 2019b) toward (a) supporting university students in understanding and thinking about their European identity and values and (b) increasing students’ awareness of the complexity of European identity and attitudes about European identity. The game was designed to help students to explore their perceptions about European identity, to develop a more detailed understanding of European identity and to promote critical thinking about their own views concerning European identity, the views of others, and the wider societal consequences.

Quantitative data regarding Greek university students’ attitudes to and perceptions of Greek and European identity, before and after playing the game, were collected. Students’ performance in the experimental game group was compared with that of a control group who engaged with the same knowledge and values content in a more traditional way, based on tutors’ lecture and students’ guided discussion. The findings showed that both instructional interventions were effective in enhancing students’ positive attitudes but in different aspects of European identity.

## Background of the Research

### European Identity and Perceptions

All educational delivery, gamed-based or traditional, must be grounded on a solid academic base (Boyle et al., 2021). However, the delivery of educational content on the topic of European identity is challenging due to the fluid and complex nature of the concepts involved. There are a number of studies illustrating that there is no clear agreement on what European identity actually is (see, for example, Kohli, 2000; Fligstein et al., 2012). It is clear that geographical location defines the EU to some extent, in the same way that it is an important aspect of national identity. However, national identity goes beyond geography and is often viewed as part of our social identity which explains belonging in terms of strong feelings that people have about being part of a group in terms of in-groups and out-groups (Tajfel and Turner, 2004). People feel pride about belonging to a group, and this sense of belonging, or not belonging, is clearly evident with European identity, where citizens’ views of the EU are very strongly felt in either supporting or opposing the EU and feeling a conflict (or not) between an EU identity and their national identity (Van der Zwet et al., 2020).

There are many different aspects of European identity. In 1993, the Maastricht Treaty established the current form of the EU and also created a firm relationship between member states, built around social, political and economic frameworks. Furthermore, the Treaty also introduced European citizenship, allowing citizens to reside in and move freely between Member States. European identity is often presented as having a strong civic component (Bruter, 2004) and Habermas (p. 7 in Mendez and Bachtler, 2016) argued that it must rest on “constitutional patriotism,” a form of civic identity behavior emphasizing democratic citizenship as the integrative force. Distinctions between an essentialist approach to identity, driven by culture, and a constructivist approach more concerned with politics are often made (Demossier, 2007). Bruter (2004) explored what citizens actually meant by “feeling European” and found a similar distinction between civic and cultural identifiers. These early indicators already suggested geographical, social, political, economic, civic and cultural elements of EU identity. This has been reinforced by other studies that have emphasized history and religion as well as culture and tradition (Maier and Rittberger, 2008) and illustrated that widespread, and differentiated understandings of European Identity exist.

However, the diversity of the EU is both a strength and a weakness, with issues of national and European identity sometimes contributing to division, marginalization and exclusion. As noted by Negri et al. (2020), many European citizens have simplistic, but very firmly entrenched, views about the value of Europe as good (pro EU) or bad (anti EU). Moreover, a sense of identity and belonging can shift over time. In 1975 over two thirds of United Kingdom voters supported staying in the then European Economic Community, but by 2016, the majority voted to leave the EU.

What is very clear is that in recent years European Union (EU) citizens and EU member states have shown fluctuating levels of commitment to membership of the European Union and this can be firmly linked to a sense of national identity (Aichholzer et al., 2021). These varied attitudes have increasingly posed challenges to the unity of the European Union, with many member states and their citizens expressing a reduced or absent sense of European-ness (Grillo, 2007; Börzel and Risse, 2018). These challenges to EU unity were recently exemplified by Brexit, the referendum vote by United Kingdom citizens in June 2016 to leave the EU. The result of the Brexit referendum and its aftermath clearly demonstrate the highly emotional and conflict-ridden nature of a problem for which there was no easy negotiated solution (Van der Zwet et al., 2020). To address these strongly polarized views and perhaps promote increased tolerance, inclusion and a culture of acceptance, EU citizens may need to develop a more widely considered, mature and nuanced understanding of both their national and European identities, that considers the validity of others’ points of view and attitudes.

### Game-Based Learning

Serious Games (SGs) are digital games designed for serious purposes, i.e., with the objective of promoting knowledge construction and supporting learning and behavior change as a result of players’ engagement. Due to their interactive and immersive characteristics, serious games are expected to create more meaningful, enjoyable, challenging, engaging and reflective learning environments compared to regular or traditional instruction (Boyle et al., 2012). In addition to basic knowledge construction, serious games encourage students’ curiosity, challenge and immersion, and also support the development of a wide range of skills such as critical thinking, collaboration, argumentation and problem solving (Romero et al., 2015; Qian and Clark, 2016). Moreover, educational games appear to be very promising in promoting students’ motivation and engagement, and supporting their learning efforts (Boyle et al., 2016).

Serious games primarily seek to promote learning on topics within specific subject areas, usually with the aim of personal or professional development, or even changing individuals’ attitudes (Boyle et al., 2012, 2016; Lerner, 2014; Seaborn and Fels, 2015). They can provide engaging activities that promote learning in challenging content areas (Boyle et al., 2012; Yu et al., 2021). In addition, SGs may help children, and adult players alike, to develop solutions and think more creatively in solving problems. Thus, educational gaming provides active, problem-solving, situated and social forms of learning, where learners reflect on their own experiences by receiving immediate and differentiated feedback that can promote possible changes in views and behaviors. Boltz et al. (2015) pointed out that the affordances of particular digital games, such as Pope’s (2013) game “Papers Please,” can foster students’ empathy as an essential skill for twenty-first century learning. Over the past decade, serious games have been increasingly recognized as engaging and effective tools for tackling complex social problems. The key idea behind game-based interventions is to increase players’ awareness of critical or timely social issues, to provide them enhanced opportunities for reflecting on their ideas and perceptions, as well as to help players to change their attitudes and behaviors.

Recognition that games can influence behavior has been evident from the early studies that suggested that playing violent video games for entertainment could lead to increases in real world aggression (Anderson and Bushman, 2001; Anderson, 2004). Gentile and Gentile (2008) identified seven learning principles that are incorporated into violent games that lead to these increases in aggression. Over time the educational potential of games was recognized as well as their capacity as agents of behavior change in more formal settings. Serious games have increasingly been recognized as active and effective methods for tackling complex social problems and encouraging behavior change and there are now many examples of such games across many different content areas including health, politics and social science.

Social impact games are games that aim to affect the player’s perspective about a specific social issue (Ruggiero, 2015) and these are clearly relevant to the objectives of the *“RUEU?”* game. These games have been developed in various areas such as advertising, environmental awareness, culture and health as well as in many social and political domains. Game-based interventions are reported in a wide range of socio-political domains for supporting attitude and/or behavior change in an intended manner. For example,

• to change or reinforce attitudes with respect to social or political causes, such as “*The Howard Dean for Iowa”* Game (Bogost and Frasca, 2007).
• to encourage behaviors that benefit society, like recycling in the case of the mobile game “*Gaea”* (Centieiro et al., 2011).
• to bring together contemporary issues such as fake news and social media misinformation, as the “*Bad News”* online game (Roozenbeek and van der Linden, 2019).
• to help players undertake the role of a border control official, through a process of psychological and physical self-modification using the puzzle-based game “*Papers, Please,”* in order to experience the cultural logic of the contemporary globalist paradigm (Kelly, 2018).
• to increase affective learning and attitude toward homelessness (Ruggiero, 2015).
• to cultivate healthy lifestyle and physical activities (Alamri et al., 2014; DeSmet et al., 2014; van’t Riet et al., 2014).
• to promote self-protection/caring, like the reduction of substance abuse and HIV risk (Fiellin et al., 2014).
• to cultivate empathy toward patients with chronic pain (Tong et al., 2020).

Although there is a burgeoning literature on social impact games, research findings regarding the effectiveness of game-based interventions still remains limited (Ruggiero, 2015; Saleme et al., 2020). However, the emerging results are promising with Johnson et al. (2017) concluding, on the basis of their review, that well-designed serious games are shaping human behavior, attitudes and cognition regarding energy consumption and related concerns. van’t Riet et al. (2018) reported that educational games can promote positive attitudes to refugees, like empathy and altruistic behaviors. Game-based interventions can induce and support environmentally friendly attitudes and behavioral change regarding environmental sustainability (Janakiraman et al., 2021).

In his book “Persuasive Games” Bogost (2010) introduced the innovative thinking behind games that use modeling of messages and social activities as a key design principle. Bogost developed these games in the fields of politics, education and advertising. Bogost makes the interesting point that, just as verbal rhetoric provides a powerful means of presenting information linguistically with the aim of influencing other people’s opinions, so persuasive games provide a procedural rhetoric, a genuinely new way of presenting information and arguments in an active, visual way that provides unique opportunities to actively persuade and influence people. Subsequently Bogost et al. (2010), coined the term “*newsgame*” which they defined as “a broad body of work produced at the intersection of videogames and journalism.” Ideas about newsgames influenced the journalist role-play narrative used within the “*RUEU?*” game.

## Objective and Research Questions

European identity is a complicated issue for people to cope with in both socio-political and educational contexts. Given the complexity of the current political situation in Europe, particularly after Brexit, and the strong polarity of citizens’ views, it was thought that a game-based intervention could provide an active and engaging method for helping students to think in more constructive ways about these issues. As Jamaludin and Hung (2017) have observed, interventions based on serious games can assist when dealing with ill-defined problems. Literature review suggested that game-based interventions aiming at attitude or behavior change in socio-political domains are in their infancy (Boyle et al., 2021). In particular, there is no study examining game-based interventions on university students regarding their development of European identity.

The online game “*RUEU?*” was designed with the aim of creating an educational platform that will help higher education students across Europe to develop a firmer and clearer understanding, not only of their own national and European identities and values, but also those of others (Boyle et al., 2019b). Since European identity is a very sensitive topic, the game was not intended to make players “more European” but to encourage them to consider and reflect upon their views concerning national and European identity. More specifically, while individuals navigate around the game they should become better informed about the EU, increase their awareness about European identity and their understanding of the complexity of European issues, and their importance in different situations and social contexts. Becoming better informed may change attitudes, but it was not the aim of the game to make players more pro or anti EU.

A discussion of the rationale underpinning the “*RUEU?”* game design process is out-with the scope of this paper (see Boyle et al., 2021 for a detailed discussion). The research presented in this paper explores the impact of using the “*RUEU?*” game on students’ attitudes regarding European identity. Responding to the research needs of exploring game-based interventions in higher education, in particular regarding socio-political issues, the objective of this particular study was to examine whether the “*RUEU?”* game was effective in (a) supporting students’ exploration and understanding of their European identity and values and (b) changing students’ awareness, attitudes and behaviors about European identity. Specifically, the following research questions were addressed:

• To what extent are students’ attitudes toward European identity affected after playing the game?
• What are the differences in students’ attitudes toward European identity comparing the results before and after the intervention in the control and the game groups?

## Research Methods

### The Sample

The selection of Greek university students as participants in this study was directed by the assumption that it could be of interest to investigate young citizens, who were growing up during the last decade. The socio-political environment during this period forced many people in Greece to be extremely divided between anti- European and pro-European attitudes.

A convenience sampling method was applied and a total of 92 undergraduate social science students, from a Greek University, agreed to participate in this exploratory study. All participants were exposed to the same environment in the University. During the previous semester, many of them were enrolled in courses like Social Policy, Political Studies, Institutions and Organization of the European Union, Contemporary History (Europe and the World), and Migration Policy. Among participants 18 students were male, 73 female and 1 student preferred not to report any gender.

With regards to the participants’ age, 56 students were 18–23 years old while 36 reported age greater than 24 years. In terms of students’ employment status, 32 had a fulltime job, 22 reported that they were working part time and 38 students were unemployed.

### Design and Procedure

A quantitative quasi-experimental design was used, where the participants were randomly assigned to the treatment groups, i.e., the control group (*n* = 46) and the experimental (game) group (*n* = 46). To examine possible differences in students’ self-reporting regarding their perceptions, feelings and attitudes about European identity were recorded and compared, before and after the instructional intervention. The results of the pre-test were used to ensure that the control and the experiment groups were equivalent, in terms of students’ prior perceptions about European identity.

Participants in both groups completed the same online questionnaire in the computer laboratory at the University and post-questionnaire completion was conducted 1 week after the intervention.

A similar procedure, which included three phases, was used in both the control and game groups: (a) introduction, (b) educational intervention, and (c) conclusion. Table 1 presents the timeline as well as students’ and tutors’ activities along the research and the instructional intervention in the two groups.

**TABLE 1:** Research and intervention procedure.

Phase	Control group	Game group
Pre-test	Students responded to the online questionnaire	Students responded to the online questionnaire
Introduction *(15 min)*	Tutor’s brief presentation of • the topic “European identity” • the objectives of the instructional intervention	Tutor’s brief presentation of • the topic “European identity” • the “RUEU?” game and the objectives of the intervention • how students’ are expected to explore and reflect on the game scenarios
Educational intervention *(90 min)*	• Tutor-guided, open discussion about themes related to European identity and European Union • Ideas interchange and interaction among students	• Students’ familiarization with the “RUEU?” game • Students’ exploration of game scenarios and reflection on themes related to European identity and the European Union • Every student provided, through the game, a structured report presenting important aspects of the European debate.
Conclusion *(15 min)*	Conclusion and debriefing	Conclusion and debriefing
Post-test	Students responded again to the same online questionnaire	Students responded again to the same online questionnaire

### The Game-Based Intervention

The students in the game-based group participated in the 2 h session in a computer laboratory. Every student worked individually on a desktop PC and had the opportunity to familiarize themselves with, explore and play the game. The students had the opportunity to play through the game scenarios and to explore the varied themes regarding EU identity. While playing the game, students were asked to think about and reflect on the game scenarios and the game content.

**FIGURE 1:**
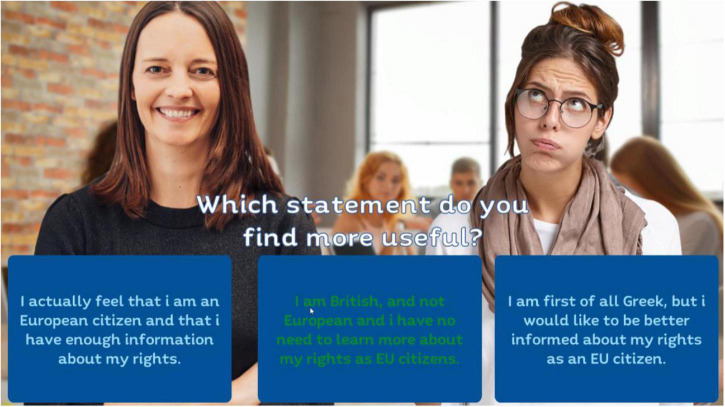
An example of statements within the “RUEU?” game.

The educational idea behind the game was to engage students in exploring their own views and biases (conscious or unconscious) about European identity, to increase individual awareness and understanding of the complex nature of Europe and the EU, emphasizing the contrasting, contradictory and frequently conflict-ridden nature of different people’s views, and to challenge them about their attitudes and prejudices in tackling problem solving tasks involving national and European identity. To achieve this, the game includes activities that require players to encounter differing views about European identity and values and to engage in tasks requiring higher-order thinking skills, such as classification, analysis, synthesis, evaluation and judgment in making decisions about the material. Figure 1 shows an example of evaluation of statements from the Discussion tool for the game.

**FIGURE 2:**
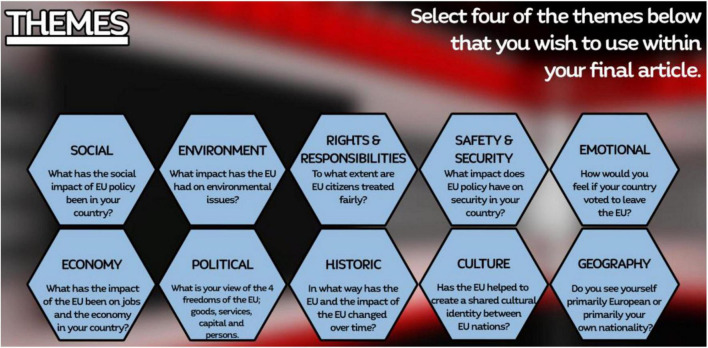
Game themes of European identity.

Based on the literature review and user requirements analysis, the content of the “*RUEU?*” game was operationalized as a multi-component construct where 10 key components (themes) were selected to reflect the breadth and complexity of factors underlying European identity: Social, Environmental, Rights and Responsibilities, Safety and security, Emotions about the EU, Jobs and Economy, Political, History, Culture, and Geography (Figure 2). Statements about these topics are incorporated into the remarks and responses made by the non-player characters (NPCs) in each scenario and in all the activities in the game.

To provide a rationale for the tasks that players are asked to do, the “*RUEU?”* game adopted the narrative of a research journalist, working on a local newspaper. The journalist’s brief is to carry out a number of assignments reporting on various issues that local readers of the newspaper are struggling with relating to European identity and values. When the player begins his task as a journalist, he is directed to a map of Europe showing five scenarios each located in a different country, that represent the journalist’s five assignments. Each gaming scenario addressed varied issues and topics that were identified as relevant to shaping the then-current views of European identity (Sim et al., 2021): (a) Brexit, (b) Freedom of movement and rights of EU citizens to work across Europe, (c) Immigration and rights of Immigrants, (d) Who is European anyway?: the differing attitudes toward Europe between EU nations, and (e) Changes in European identity over time.

Each scenario includes a number of activities that are typical of a journalist’s day-to-day tasks in tracking down material to write a story and these are presented as tools. The game tools that help players in their effort of collecting, summarizing and organizing statements are (a) the Mobile phone chat, (b) the Interview tool, (c) Discussion tool, (d) Newsflash tool, and (e) the Final assignment tool. The activities all required players to encounter differing views about European identity and think more critically about the content implemented, their conversations with a range of stakeholders (non-player characters), their exploring of audio and video extracts as well as twitter accounts in order to develop statements and comments uttered by the game tools and compile the material for his/her report.

**FIGURE 3:**
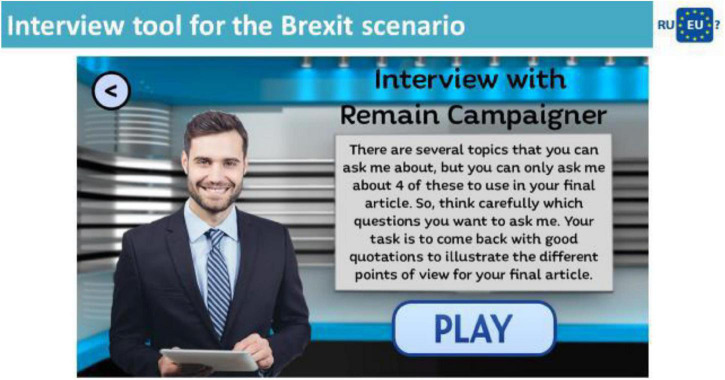
A screenshot of the Interview tool with the Brexit scenario.

**FIGURE 4:**
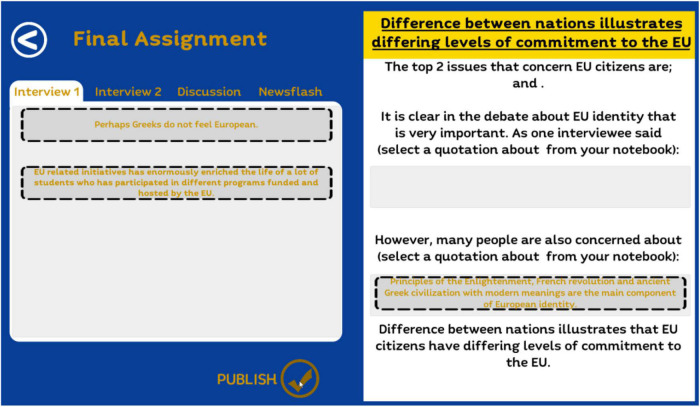
Final assignment and game tools.

For example in the Interview tool (Figure 3) the player takes part in interviews with leading representatives of (a) a pro EU and (b) an anti EU campaign. When he presses “play” the player is presented on screen with the 10 themes shown in Figure 2 and he has to select the 4 issues that he would most like to ask the pro and anti EU representatives about. When he has asked the question, the player is then shown the NPC’s answer to the selected issues. For example the NPC might state that: “Freedom of movement has helped to create a much richer and more diverse society” or “If we vote to leave the EU, my company will go down the tubes.” Each statement made by a NPC is coded with respect to whether it is pro or anti EU and which theme it represents. For example the first statement is coded as a pro EU “social” statement and the second as an anti EU “jobs and economy” statement. The selected statements are then transferred to the player’s notebook where they are available for the player to choose in “writing” his article in the Final Assignment tool (Figure 4).

The final objective of playing the game is to help the player to present a structured report that explores aspects of the European debate. The material for this Final Assignment is based on the player’s selections and choices in the Interview tool, the Discussion tool and the Newsflash tool. The players’ selections are categorized and organized into a draft article to be “published” in the newspaper. As a good journalist, pros and cons from various resources should be clearly reflected in the students’ articles.

### Control Group Intervention

The students in the control group participated in a 2 h lecture-based classroom intervention about European identity. The intervention was designed as an in-person classroom session, organized into the three stages, where instead of the game intervention, students took part in a tutor-guided session involving open discussion, brainstorming, argument and short debates (lasting 90 min) focused on critical components of European identity extracted from the 5 scenarios and 10 themes of the “*RUEU?”* game content.

### The Questionnaire

An online anonymous questionnaire was the instrument used to record students’ European identity perceptions and attitudes in both, pre- and post- intervention phases (Supplementary Appendix). Students were also asked to provide demographic information such as their gender, age and work status.

The development of the instrument was based upon the existing literature with most questions based on the “Eurobarometer” survey, a questionnaire that assesses how popular the EU is, and included questions about the extent to which respondents think of themselves as citizens of Europe (Eurobarometer, 2018). The statements were positively worded in order to represent the anti- European (value = 1 and 2) and pro-European (value = 4 or 5) extremes. On the whole, higher scores meant greater attachment/belonging to Europe etc.

The participants were asked about their personal meaning and understanding of the notion of “European identity” along five factors: (a) sense of geographical belonging, (b) thinking as European, (c) attachment to the European Union, (d) balance between national and European identity, (e) sense of pride in belonging to national country and Europe. The statements regarding the balance between national and European identity were expressed in the “Moreno” question (Moreno, 2006) which is a very simple, one item measure that reflects an individual’s perception of the balance between his/her national and European identity, emphasizing which is relatively more important. Scores lower than 3 indicate that individuals would describe themselves as more their own nationality than European, while scores higher than 3 indicate that individuals would describe themselves as more European than their own nationality.

## Results

### Geographical Belonging

Our analysis revealed three main groups of students with regards to their feelings of geographical belonging. No statistically important difference was recorded, in the control and the game groups, before and after the intervention.

In the first group of students we classified, approximately one third of the participants reported their country as the main geographical area of belonging (35.9% of the participants and 33.7%, respectively, before after the intervention). On the other hand, Europe was the choice of 1/4 of the students (23.9% before and 25% after the intervention). Four out of ten students in the sample, before and after the intervention as well, appeared to adopt locality in their view of belonging, in terms of the region or the town where they are living.

It was quite interesting that Europe was selected by the majority of students as the next most important geographical area in both research phases, i.e., before (26.1%) and after the intervention (27.2%). According to their choices, the participants were actually divided to three similar groups; i.e., the country as a whole (23.9% before and 22.8% after the intervention), the country region of living (25 and 21.7%), and the town of living (19.6 and 25%). No statistically significant difference was recorded among the control and the game groups.

Interestingly age was a critical factor affecting students’ views of geographical belonging. A Mann-Whitney *U*-test showed that students older than 24 years appeared to choose Europe as their primary geographical area of belonging (*U* = 652.5, *p* = 0.003), while those in the younger age group were divided between the national and local perceptions of geographical belonging.

### Students’ Perceptions of European Identity Before the Intervention

Table 2 presents the descriptive statistics regarding students’ responses to the ten European identity questions, before the instructional intervention (*N* = 92). In general, the participants were positive about most of the aspects of European identity under investigation. The majority of the students in the sample appeared to be thinking as both Greek and European citizens. As expected, their feelings were stronger about their own country, with higher scores for the questions on attachment to their own country (Q7) compared with the question on attachment to Europe (Q4). However, they felt a sense of pride both in belonging to Greece (Q9) and in being an EU citizen (Q10) and they also believed that what happens in the EU affects them, with a high score on that question (Q6).

**TABLE 2:** Students’ perceptions of European identity before the intervention (*N* = 92).

Item	Mean	*SD*
Q1. Does the term “European identity” mean anything to you?	3.98	1.20
Q2. Do you ever think of yourself as a citizen of Europe?	3.22	0.80
Q3. Do you ever think of yourself as not only (your nationality) but also European?	3.18	0.84
Q4. How attached or close do you feel to the EU?	3.49	1.26
Q5. How much does being an EU citizen have to do with how you feel about yourself in your day-to-day life?	3.28	1.35
Q6. How far do you feel that what happens in the EU in general has consequences for people like you?	4.25	1.07
Q7. How attached or close do you feel to your own country?	4.09	1.10
Q8. In this list, can you say how you would describe yourself?	2.43	0.91
Q9. Do you feel a sense of pride at belonging to your own country?	4.13	1.13
Q10. Do you feel a sense of pride at being an EU citizen?	3.96	1.13

With regards to the Moreno question (Q8), the mean score of 2.43 is low, indicating that, despite tending to agree that they sometimes think of themselves “as a citizen of Europe” (Q2) and that they think of themselves as not only Greek but also European (Q3), participants still tended to describe themselves as “More Greek than European.” The majority of the students (6 out of 10) prioritized the Greek nationality while a large group of their colleagues (3 out of 10) perceived the Greek nationality and European identity to be of equal value. On the other hand, 1 out of 10 students prioritized their European nationality.

### Students’ Perceptions of European Identity After the Intervention

Table 3 summarizes students’ mean ratings for each of the EU identity items following the game based and control interventions, showing also the items with statistically significant differences from pre to post intervention. Both forms of the instructional intervention increased students’ ratings, but differed in which items were impacted. Wilcoxon Signed-ranks tests were used to examine pre-post differences for the different items.

**TABLE 3:** Students’ perceptions of European identity after the intervention.

Item	Control group (*N* = 46)		Game group (*N* = 46)	
		
	Mean	*SD*	Mean	*SD*
Q1. Does the term “European identity” mean anything to you?	4.35*	0.80	4.17	1.00
Q2. Do you ever think of yourself as a citizen of Europe?	3.35	0.60	3.30	0.59
Q3. Do you ever think of yourself as not only (your nationality) but also European?	3.33	0.60	3.30	0.73
Q4. How attached or close do you feel to the EU?	3.85	1.01	3.83*	1.10
Q5. How much does being an EU citizen have to do with how you feel about yourself in your day-to-day life?	3.80*	1.19	3.41	1.2
Q6. How far do you feel that what happens in the EU in general has consequences for people like you?	4.20	1.05	4.22	0.92
Q7. How attached or close do you feel to your own country?	4.17	0.90	3.98	0.88
Q8. In this list, can you say how you would describe yourself?	2.43	0.69	2.83*	0.90
Q9. Do you feel a sense of pride at belonging to your own country?	4.04	1.12	4.09	1.01
Q10. Do you feel a sense of pride at being an EU citizen?	3.96	1.01	3.89	1.04

*Statistically significant difference (p < 0.05).

While both groups increased in their ratings of attachment (closeness) to the European Union following the interventions, as shown in Table 3, this increase was only statistically significant for the game-intervention group (*Z* = 2.252, *p* = 0.024). There was also an increase for the game-based group in their ratings on the Moreno question, showing a shift toward a more balanced recognition of their European identity as well as their Greek identity (*Z* = 1.946, *p* < 0.05). A significant difference was also identified post intervention between the experimental and control group with regards to the Moreno question. Using Wilcoxon Signed-ranks test (*Z* = 5.483, *p* < 0.01), the students in the game group appeared to move toward a more balanced perception of their European and national identity (*M* = 2.83; *SD* = 0.90) compared to their fellows in the control group (*M* = 2.43; *SD* = 0.69). However, it is important to notice that both before and after the interventions, means were less than 3, indicating an enduring tendency for individuals to describe themselves as more Greek than European. Before the intervention, approximately one third of the students in the two groups, reported that they feel equally Greek and European.

Even before the intervention, students in both groups agreed that the term “European identity” meant something to them. Following the interventions this increased for both groups suggesting that the interventions were successful in helping students to understand European identity. However, this difference was only statistically significant for the control group (*Z* = 2.156, *p* = 0.031). Similarly, pre-intervention both groups tended to agree that being an EU citizen affects them in their day-to-day life. Agreement increased for both groups post intervention, but the increase was only significant for the control group (*Z* = 2.714, *p* = 0.007).

### The Role of Students’ Age

No statistically significant differences in attitudes to the EU were identified with respect to students’ gender. However, age appeared as a critical factor in determining students’ perceptions of EU identity in the game intervention groups. Table 4 summarizes the findings concerning the two age groups in our analysis, before and after the intervention. Mann-Whitney *U*-tests revealed that statistically significant differences were found following the interventions, between the younger students (aged 18–13 years) and their older fellows (with age greater than 24 years) with respect to both European and national items. More specifically, the older students were more pro-European with respect to: (a) European citizenship (*U* = 786, *p* = 0.041); (b) attachment to the EU (*U* = 784, *p* = 0.050) and (c) the effect of being an EU citizen to day-to-day life (*U* = 739, *p* = 0.021). However, they also had (d) stronger feelings of attachment to Greece (*U* = 759, *p* = 0.027); and (e) a stronger sense of pride in belonging to Greece (*U* = 629, *p* = 0.001).

**TABLE 4:** Students’ perceptions of European identity after the intervention (age groups).

Item	18–23 (*N* = 56)		24 + (*N* = 36)		p
		
	Mean	*SD*	Mean	*SD*	
Does the term “European identity” mean anything to you?	4.23	0.79	4.31	1.06	
Do you ever think of yourself as a citizen of Europe?	3.23	0.57	3.47*	0.61	0.041
Do you ever think of yourself as not only (your nationality) but also European?	3.25	0.69	3.42	0.60	
How attached or close do you feel to the EU?	3.68	1.06	4.08*	1.00	0.050
How much does being an EU citizen have to do with how you feel about yourself in your day-to-day life?	3.38	1.27	3.97*	1.06	0.021
How far do you feel that what happens in the EU in general has consequences for people like you?	4.05	1.09	4.44	0.74	
How attached or close do you feel to your own country?	3.93	0.91	4.31*	0.82	0.027
In this list, can you say how you would describe yourself?	2.59	0.85	2.69	0.71	
Do you feel a sense of pride at belonging to your own country?	3.79	1.14	4.50*	0.74	0.001
Do you feel a sense of pride at being an EU citizen?	3.77	1.08	4.17	0.88	

*Statistically significant difference (p < 0.05).

Employment status was also revealed as a factor related to students’ positive perceptions and attitudes about European identity. Compared to their fellows who are unemployed or working part time, the students who were working fulltime reported higher rates along all items in scale, before and after the intervention. Since the various socio-economic factors appear to affect European identification (Lam and Katona, 2018), this finding needs further exploration in broader contexts and across European countries as well.

## Discussion and Conclusion

In this paper we presented an experimental study exploring the effects of a game-based intervention on higher education students’ understanding and perceptions of socio-political issues regarding European identity. With regards to the first research question, the findings showed that playing the game did significantly change certain aspects of students’ attitudes to European identity. After playing the game students reported feeling more attached to Europe and their responses on the Moreno question also shifted significantly toward a more balanced recognition of their European identity as well as their Greek identity. However, it is important to note that in both groups, before and after the interventions, means on the Moreno question were less than 3, indicating an enduring tendency for individuals to describe themselves as more Greek than European. The results suggest that game-based interventions can be integrated into higher education contexts, not only with the aim of delivering academic content, but also in promoting students’ reflection at both attitudinal and emotional levels.

Interestingly the control (tutor guided) intervention also led to changes in aspects of students’ European identity, although on different questions to the game. Following the intervention, participants in the control condition were more likely to agree that the term “European identity” meant something to them and that being an EU citizen affects them in day-to-day life. In fact, post intervention, the control group had significantly higher means for agreeing that the term “European identity” meant something to them than the game group. This showed that classroom discussion, the interchange of ideas among students and debate methods were also beneficial in changing students’ understanding of European identity and suggests that guided classroom discussions may be more efficient at increasing understanding of European identity than the game based approach.

The changes in students’ responses from before to after the interventions were not large but generally suggested that learning about European identity gives students a more positive awareness of the EU and perhaps counters some of the negative perceptions of “Europe” in sections of the mass media. The fact that, in some cases, these changes occurred regardless of whether students played the game or received a traditional lecture based on the same materials suggested that gaming is not necessarily a substitute for more traditional learning. Since the students, who participated in the study, had no previous learning experience based on educational games, further research is necessary to examine more engaging, effective and integrative ways of using serious games in higher education contexts.

The findings presented confirm that national and European identity are not contradictory: most citizens can feel allegiance both to their own nation and to the EU. These observations are consistent with Risse’s marble cake model of identity, where the various components of an individual’s identity cannot easily be separated; rather they influence each other, mesh and blend (Risse and Grabowsky, 2008). Further investigations are necessary to explore the multifaceted aspects of and the factors that determine young Europeans’ attitudes and perceptions of EU identity.

Age was also found to impact on European identity with older students, in the 24 plus age bracket, having stronger pro-European feelings with respect to European citizenship and attachment to the EU and also agreeing more strongly that being an EU citizen influenced how the felt about themselves in their day-to-day life. However, the older students also had stronger attachment to and pride in Greece. Our results with the Greek students contrast with those mentioned in the introduction that suggested that younger groups had more positive attitudes to the EU that older groups. Our results may reflect that the older students had more experience of thinking about these identity issues. However, it is possible that the weaker pro EU feelings of the younger group are due to the fact that this cohort was growing up during the time of the financial crisis in Greece, as well as the crises with immigrants. These younger students were possibly exposed to views that the EU did not help them during these crises.

As Brexit demonstrated, changes take place over time in citizens’ attitudes to the EU. However, a recent Eurobarometer survey (Eurobarometer, 2021), carried out in June-July 2021, found that attitudes toward the EU remained positive and broadly stable. Optimism about the future of the EU has reached its highest level since 2009 and trust in the EU remains at its highest since 2008. Support for the euro remains stable at its highest since 2004. These positive attitudes to and optimism about the EU are consistent with the more positive views of the older group of students in the current study.

Undoubtedly, European identity is a complex, multi-faceted and inherently imprecise concept related to a range of socio-political and psychological factors. Addressing this topic in educational practice, particularly with respect to higher education students, constitutes a complex and open problem for research. The “*RUEU?*” game recognized the complexity of identity, and did not reduce our understanding to simple binary constructs, such as pro or anti, that simplify and limit their meaning. Consistent with De Cillia et al.’s (1999) claim that: “the discursive construction of national identities is a multidimensional phenomenon” (p. 170), the design of the “*RUEU?*” game adopted a multidimensional operationalization of EU identity in terms of the 5 scenarios and 10 themes. While this emerged from the research carried out in the literature review and user requirements analysis carried out for the game, it could of course be questioned whether significant components were missed.

The game was innovative in presenting different elements of a contentious construct via statements and comments made by NPCs and requiring players to carry out higher level thinking tasks where they had to categorize, sort, select, prioritize and evaluate these statements. By taking part in the tasks, players’ awareness of their own views and the views of others was increased and they were alerted to the complexity of the construct. It is envisioned that these innovative ideas could be applied to or adapted to address many other divisive social issues, by offering a controlled environment where differing attitudes and their associated behaviors and consequences can be explored and evaluated. Issues might include gender identity, climate change, social care etc. Game-based interventions in various socio-political domains aiming at students’ reflection and supporting attitude or behavior change constitute an open field for research and development (Ruggiero, 2015; van’t Riet et al., 2018; Janakiraman et al., 2021).

### Limitations and Future Research

The choices selected by players in the game scenarios were personal preferences, intended to reflect their views of the key issues and most relevant statements about EU identity. In that sense there were no right or wrong answers, so it was not possible to give feedback based on whether responses were correct. With sufficient data about players’ selections of themes etc., a future version of the game could include more feedback about other people’s responses. It would also be good to include more activities where the player suffered the consequences of his actions. To make it more game-like, more use could be made of traditional game features such as rewards, and constraints on progress through the game, such as only being able to tackle specific tasks once others have been completed.

A second limitation is related to the participants in this study who were undergraduate social science students at a Greek University. They had the opportunity to attend a number of courses relating to many political, social, historical and cultural issues around which the European identity is supposed to be shaped. Therefore, they were more sensitive to/or positive about the critical issues determining European identity than other students and young people. In addition, some students had also experience of international studies as Erasmus + students abroad and this may affect their positive attitudes about Europe and EU identity, as pointed out by Jacobone and Moro (2015).

In addition, the Greek context is another limitation that did not allow our results to be generalized to students from other countries. It could be very interesting to explore possible differences in HE students’ attitudes over time, for example in Britain after Brexit, in secondary education students etc.

Given the context-specific nature of the European identity, further research, ideally longitudinal in nature, is necessary to shape the possible trajectories that young Europeans follow toward developing their national and European identity as well as the educational practices that promote students reflection, rethinking and behavioral change with regards to the key factors intervening. In this perspective, combining both quantitative and qualitative data could further support the findings of the present survey and shed light into the complex factors that affect students’ and young adults’ feelings and perceptions of EU identity.

Importantly for research on games the current study adds to the evidence that games can be used to change behavior and attitudes in socio-political domains. Overall our findings showed that both instructional interventions were effective in supporting behavior and attitude change in the sensitive area of European identity. It also suggested that the game and the traditional teaching interventions worked on different aspects of this complex construct. Although creating serious games like this is costly and time consuming, serious games are very much in line with current educational trends in online education. We would even argue that creating games such as this can help us to further understand complex social issues.

## Data Availability Statement

The raw data supporting the conclusions of this article will be made available by the authors, without undue reservation.

## Ethics Statement

This research project was deemed exempt by the Research Ethics Committee of the University of Peloponnese because it does not involve clinical data measurement. Written informed consent to participate in this study was provided by the participants through the online questionnaire.

## Author Contributions

AJ, EB, PT, and ML contributed to the conception and the design of the study. AJ and PT conducted the instructional interventions and organized the database and performed the statistical analysis. AJ and EB wrote the first draft of the manuscript. PT and MT revised sections of the manuscript. All authors contributed to manuscript revision, read, and approved the submitted version.

## Conflict of Interest

The authors declare that the research was conducted in the absence of any commercial or financial relationships that could be construed as a potential conflict of interest.

## Publisher’s Note

All claims expressed in this article are solely those of the authors and do not necessarily represent those of their affiliated organizations, or those of the publisher, the editors and the reviewers. Any product that may be evaluated in this article, or claim that may be made by its manufacturer, is not guaranteed or endorsed by the publisher.
