# Supplementary material for: Exploring the Impact of the “RUEU?” Game on Greek Students’ Perceptions of and Attitudes to European Identity
Source: Front Psychol. 2022 Mar 16;13:834846. doi: 10.3389/fpsyg.2022.834846 (PMC8966699; doi:10.3389/fpsyg.2022.834846)
Supplement: Supplementary file 1 [file Data_Sheet_1.docx]

**Appendix A. The questionnaire**

**Demographic information**

Age

Gender

National identity (Greek)

Country of Residence (Greece)

Area of HE study (Social Sciences)

Work status

**Items**

**Which of these geographical groups would you say was the most important to you in terms of where you feel you belong?**

1. The locality or town where you live
2. The region or country where you live
3. Your country as a whole
4. Europe
5. Don’t know

**And the next most important?**

1. The locality or town where you live
2. The region or country where you live
3. Your country as a whole
4. Europe
5. Don’t know

**Does the term ‘European identity’ mean anything to you?**

1. Not at all
2. Not very much
3. Don’t know
4. Yes, somewhat
5. Yes, a lot

**Do you ever think of yourself as a citizen of Europe?**

1. Never
2. Don’t know
3. Sometimes
4. Often

**Do you ever think of yourself as not only (your nationality) but also European?**

1. Never
2. Don’t know
3. Sometimes
4. Often

**How attached or close do you feel to the EU?**

1. Not at all attached/ not at all close
2. Not very attached/ not very close
3. Don’t know
4. Fairly attached/ fairly close
5. Very attached/ very close

**How much does being an EU citizen have to do with how you feel about yourself in your day-to-day life?**

1. Not at all
2. Not very much
3. Don’t know
4. Somewhat
5. A great deal

**How far do you feel that what happens in the EU in general has consequences for people like you?**

1. Not at all
2. Not very much
3. Don’t know
4. Somewhat
5. A great deal

**How attached or close do you feel to your own country?**

1. Not at all attached/ not at all close
2. Not very attached/ not very close
3. Don’t know
4. Fairly attached/ fairly close
5. Very attached/ very close

**In this list, can you say how you would describe yourself?**

1. My nationality only
2. More my own nationality than European
3. Equally my own nationality and European
4. More European than my nationality
5. European only

**Do you feel a sense of pride at belonging to your own country?**

1. Not at all proud
2. Not very proud
3. Don’t know
4. Fairly proud
5. Very proud

**Do you feel a sense of pride at being an EU citizen?**

1. Not at all proud
2. Not very proud
3. Don’t know
4. Fairly proud
5. Very proud
